# Understanding health inequalities through a practice‐oriented ‘capabilities’ perspective. Motherhood and leisure time physical activity

**DOI:** 10.1111/1467-9566.13585

**Published:** 2022-11-15

**Authors:** Fiona Spotswood, Lauren Gurrieri

**Affiliations:** ^1^ University of Bristol Business School, University of Bristol Bristol UK; ^2^ School of Economics, Finance and Marketing RMIT University Melbourne Victoria Australia

**Keywords:** capabilities, inequalities, mothering, physical activity, practice theory

## Abstract

In this article, we explore how a capabilities perspective can advance practice theoretic conceptualisations of persistent health inequalities. Specifically, we seek to understand the capabilities necessary for recruitment to leisure time physical activity (LTPA) practices by low SES mothers, a group traditionally excluded from LTPA. Our study illuminates that mothers living a life of social disadvantage face difficulties in becoming recruited to LTPA practices despite, in many cases, the availability of elements required for performance. We identify that temporal, support and energy capabilities are necessary for low SES mothers to become recruitable to LTPA. The dispossession of these capabilities signals inequalities in the constellation of practices that configure this group’s lived experiences, in turn giving rise to practice absence and further consolidating patterns of inequality. We offer a framework of practice capabilities and health inequalities to guide future practice‐oriented scholarship in the sociology of illness and health, which signals how capabilities may enable or constrain recruitment to health‐promoting practices, give rise to inequalities and condition the possibility of practice absence.

## INTRODUCTION

Practice theory accounts of health and illness emphasise that health outcomes emerge from the way that everyday life is organised through the intersection of dynamic practices and their characteristics rather than from individuals’ flawed decisions and personal deficits (Blue et al., 2016; Keane et al., 2017; Maller, 2015; Twine, 2015). This view foregrounds how collectively understood, shared practices, constituting elements comprising meanings, materials and competences (Shove et al., 2012), are reproduced by practitioners through their largely habituated, unreflexive performances in everyday life. These performances are enacted broadly in line with the practice blueprint (Molander & Hartmann, 2018), and in the context of interconnected, coordinated nexus of practices that shape the way performances become meaningful and evolve. For example, studies have illustrated how problematic practices like smoking and drinking alcohol become meaningful through their social, temporal and material location with other practices that matter in everyday life (Ally et al., 2016). Similarly, studies have illuminated the temporal characteristics of mothering practices that readily push out everyday leisure time physical activity (Spotswood et al., 2021). From this perspective, unhealthy body mass can be considered an outcome of recruitment to a particular set of practices that become inscribed in bodies (Maller, 2017), and obesity as a societal problem has been conceptualised as a ‘trace’ of large, interconnecting and changing complexes of practices that become embodied and passed on intergenerationally (Blue et al., 2021, p. 9).

There are established advantages to conceptualising health in practice‐ontological terms (Cohn, 2014), particularly given the notable limitations of policy approaches that focus on providing more information to people to support individual attitudes and choices or on investing in infrastructure to increase access and availability to places to exercise (Nettleton & Green, 2014). These individualist policies rely on assumptions of individual sovereignty, often responsibilise individuals to make different, ‘informed’ and voluntary choices (Delormier et al., 2009), and fail to disrupt the socio‐structural conditions underpinning the unequal constraints on the way healthy behaviours can become part of the fabric of everyday life. The focus on characteristics of configurations of everyday practices and their elements shifts focus away from individual or structural deficits, affording consideration of how policies might intervene at a practice level (Maller, 2015).

However, a significant gap in existing empirical practice‐oriented health research is the lack of attention given to theorising unequal patterns of practices in relation to health and how targeted interventions might be configured within a social ontology of practices. This is problematic given that inequalities are a key concern for public health (Bergey et al., 2022) and that patterns of practice and practice absence characterise the national picture of health. Indeed, many practice‐oriented health studies present the trajectories of practices as largely undifferentiated (Hennell et al., 2021; Keane et al., 2017; Twine, 2015). Halkier and Holm (2021) and Maller (2019) emphasise that practice theory has failed to adequately account for inequalities in health‐related practice performance, and Walker (2015) critiques practice theory generally for brushing over questions of social difference.

Where inequality is referenced in health‐oriented practice theoretical research, the focus tends to be on the unequal ‘availability’ of practice elements, which are necessary for practitioners to integrate in moments of practice performance (Molander & Hartmann, 2018). For example, Ally et al. (2016) identify patterns of alcohol consumption in terms of social grade and how people drink, noting that elements of practice have ‘different availability and relevance’ (p. 1577) across society. Similarly, Blue et al. (2016) argue that inequality in health is largely a matter of understanding the distribution of requisite elements:

So how is it that certain individuals become the carriers of smoking whilst others do not? Practices depend on the coexistence and availability of requisite elements—competences, materials and meanings—but these are not evenly distributed across society.

(p. 44)

This view foregrounds the practice entity, with its circulating elements that are integrated by practitioners in the moment of performance. Participation is then assumed to depend solely on the unequal distribution of competences, materials and meanings, the outcomes of unequal patterns of past practices. In this line, obesity is explained through the uneven distribution of practices that prevent obesity (Maller, 2016), like physical activity (PA) and healthy eating practices, because there is an uneven distribution of the elements necessary for their enactment (Blue et al., 2016; Warde, 2005). These elements are accrued through ongoing practice performances. In this way, patterns of socioeconomic disadvantage become cyclical because requisite elements are only made available through practices, so the possibilities of future practices are conditioned by ongoing practice performances.

This deindividualised theorisation of inequality in practice theory has enabled practice theorists to contribute to conceptualisations of intergenerational health inequality (Blue et al., 2021), yet rigorously disregards lived experiences (Molander & Hartmann, 2018) and presents recruitment as simplistic and unproblematic (Walker, 2015). Thus, it risks missing important insights into experiences of exclusion and conditions of recruitment to practices that can inform an enriched theorization of inequality within a practice‐oriented account. An alternative perspective, still within the social paradigm of practice, advances from the emphasis on practice and practice element availability to include focus on how practitioners are differently abled, and experience constraints, in approaching, accessing and integrating the elements necessary for enactment. In this line, Maller (2019, p. 91) notes that ‘abilities and capacities’ of practitioners also have a role to play in practice trajectories. However, this practitioner‐oriented perspective is undertheorised.

We draw on the capabilities framework (Sen, 2005, 2009) to theorise the way that practitioners are enabled or constrained in integrating practice elements and the way the conditions for these emerge from unequal patterns of practices characterising the everyday lives of those who experience exclusion from health‐promoting practices. The capabilities framework has been recognised as important in public health (Prah Ruger, 2010) for an understanding of inequality that is focused on the ability or freedom to access important ‘doings’ and ‘beings’, rather than solely the realisation of outcomes (Sen, 1999). Recently, it has been used in other fields as offering theoretical leverage for explaining inequality in practice recruitment (Day et al., 2016; Walker, 2013). Furthermore, it has been suggested by Halkier and Holm in this journal as a possibility for developing a ‘differentiated’ practice approach to health research by focusing on ‘the abilities to perform, or enact, practices—for example, being able to draw upon materials—competences and meanings’ (2021, p. 9).

In this article, we illuminate the capabilities necessary for recruitment to leisure time physical activity (LTPA) practices by low SES mothers in order to advance practice theoretic conceptualisations of persistent health inequality. Through our qualitative empirical investigation of how this group comes to be excluded from LTPA practices in the UK, we identify three capabilities that are necessary for their freedom to become recruitable to LTPA; temporal, support and energy capabilities. We discuss the implications of a capabilities approach to practice‐oriented health and illness research in terms of conceptualisations of practice recruitment, inequalities and absence in relation to health.

## DEVELOPING A ‘CAPABILITIES’ APPROACH FOR PRACTICE THEORY ANALYSIS OF HEALTH INEQUALITIES

This study advances from previous work that conceptualises inequality in patterns of practice performance in terms of the availability of elements necessary for enactment whilst undertheorising the resources required for accessing and integrating these. Following Warde (2005), we recognise the potential contribution of practitioner resources to the reproduction and development of practices, which is missing from the existing conceptualisations of inequality in practice theory. We recognise that many different capabilities might differentiate the plane upon which agents participate in practices because they might prevent practitioners from being recruited or might challenge the capacity for practitioners to integrate the elements necessary for practice performance; the materials, meanings and competences prescribed by the practice blueprint. Socially patterned availability of capabilities therefore places conditions on practice recruitment, which helps perpetuate the configuration of the nexus through which practices and their elements circulate or are withheld.

The ‘capabilities’ perspective refers to the capacity of individuals to perform important functionings such as ‘enjoying good health’ or ‘participating in political choices’, which will vary in value over time and depending on the value‐orientations of society (Gangas, 2016). Together, functionings comprise a valued life that provides wellbeing. Capabilities, then, structure freedoms to live the lives people value and to achieve the identities they desire (Sen, 2009).

Sen (2005) developed the capabilities perspective in order to contextualise ‘opportunity’ in the following terms:

(i) whether a person is actually able to do things she would value doing, and (ii) whether she possesses the means or instruments or permissions to pursue what she would like to do (her actual ability to do that pursuing may depend on many contingent circumstances).

(p. 153)

Thus, people with the same ‘means’ to make the most of an opportunity (such as income, wealth and other primary resources) may still have differentiated freedoms on the basis of important structuring capabilities (Sen, 2005). Income may be a capability but is contingent on other factors, such as how much money is required for mobility and participation in valued functionings, which will be greater for a disabled person. Other capabilities will be linked to specific practice domains (Walker, 2015), but may include physical and mental health, cognitive capacity, emotions, group membership and cohesion, political context and control (Nussbaum, 1992). This perspective can underpin research that explores the capabilities necessary for participation in particular fields, by particular groups, to theorise their exclusion and frame necessary intervention.

Bringing capabilities into practice theory in the context of sustainable consumption, Walker (2015) situates capabilities at the intersection of practices and practitioners, arguing that they problematise the traditionally unproblematic notion of recruitment. Practices require the integration of a prescribed but diverse range of elements according to their template (Molander & Hartmann, 2018), and some practitioners will be better equipped than others to achieve this ‘putting together’ or integration:

We might expect that some practitioners will be in a better position to integrate the necessary materials, competences and meanings that constitute a given practice and that some will be likely to do so with more success… than others.

(Walker, 2015, p. 51)

Thus, recruitment is not just contingent on available elements, which represent the required components necessary for practice enactment according to the practice blueprint. No matter how desirable a practitioner might be or how available a practice might be to recruit them, capabilities are also necessary for the integration of these elements into practice performances and therefore are important factors in understanding the trajectories that practices take. An example is normatively charged sports practices that might be understood as masculine, rendering them ‘exclusive and particular rather than inclusive and open to all’ (Walker, 2015, p. 52). Here, women may lack the capabilities to be recruited in the context of strong sociocultural expectations about ‘gender appropriate’ leisure, despite the potential availability of materials, meanings and competences and a woman’s desire to take part. Crucially, these capabilities are specific to women and require careful intervention to overcome.

Bundles of past practices, their life course and the lived experience of social disadvantage have been highlighted in existing practice‐oriented accounts of health as important for understanding health inequalities (Blue et al., 2016; Maller, 2017). A capabilities perspective provides compelling theoretical leverage for interpreting the links between past practices and future recruitment than currently exists. We argue that paying closer attention to how patterns of practices (including those promoting health) are distributed across populations and relating these to the distribution of capabilities necessary to perform them is a way that practice theories might account for health inequalities and inform intervention and policy approaches. Grounded in this practice‐based capabilities‐oriented theoretical framework, we explore the enmeshed practices that colonise the everyday lives of lower SES mothers, through which we illuminate the capabilities that condition their enactment of LTPA practices. This group is traditionally excluded from LTPA in comparison with more affluent mothers, women without children and men (Bellows‐Riecken & Rhodes, 2008; Mailey & Hsu, 2019). This study thus employs a capabilities perspective to understand how inequalities arise for lower SES mothers in recruitment to LTPA practices.

## METHODOLOGY

This qualitative research took an interpretivist approach based on principles of reflexive thematic analysis, as our methodology focuses on ‘meaning that is context‐bound, positioned and situated’ (Braun & Clarke, 2019, p. 591). Semi‐structured, in‐depth interviews were adopted for the study, focussing on the everyday lives of lower SES mothers with at least one pre‐school child. Their everyday lives represent the routinised practice performances they repeatedly enact and thus reconstitute (Will & Weiner, 2014). We particularly focussed on this group’s experiences of exclusion from, and feelings towards, leisure time physical activity (LTPA). Online in‐depth interviews (*n* = 26) were conducted between March and December 2020, at the height of the UK’s COVID‐19 social distancing measures, or ‘lockdowns’. The sample table is shown in Table 1. During this time, social marketing campaigns from the UK’s National Health Service encouraged adults to use the lockdowns as ‘time for a reset, restart, kickstart’; to get active and lose weight to better resist the ill‐effects of COVID‐19^1^. At a time when no formal LTPA opportunities were available to anyone irrespective of income, this period provided an opportunity to explore low SES mothers’ perceptions of future prospects for LTPA in the context of past experiences and entrenched routines.

**TABLE 1:** Sample table

Pseudonym	Ethnicity	LTPA (not including lockdown‐enforced inactivity)	Pre‐motherhood LTPA	Family
Josie	White British	Low	Low	Married. Lives with husband (multiple disabilities), 2 children 17m and 4 years (disabilities)
Clara	White British	Inactive	Regular	Lives with male partner, 2 children, 2 and 8.
Rebecca	White British	Inactive	Low	Married. Lives with 2 children, 3 and 15m.
Diana	White British	Inactive	Regular	Married. Lives with husband, 2 children, 4 and 10m.
Laura	White British	Inactive	Inactive	Single. Lives with 2 children, 16 and 16m. She has some physical disabilities.
Jemima	Polish	Inactive	Regular	Single. Lives with 3 children, 4, 12 and 14.
Amma	Black British	Inactive	Low	Single. Lives with 2 children 14 years and 5m (both with disabilities).
Rhoda	White British	Inactive	Regular	Single. Lives with 2 children but only 1 lives with her, 2 years.
Tia	White British	Inactive	Low	Single. Lives with 1 child, 18m.
Dora	White British	Inactive	Regular	Lives with male partner and 2 children, 14 (Special needs) and 5m.
Fran	White British	Inactive	Low	Lives with male partner and 1 child, 10m.
Vanessa	White British	Inactive	Low	Single. Lives with 2 children 2, 3 months.
Kate	White British	Inactive	Regular	Single. Lives with 2 children, 5 and 2.
Jenny	White British	Inactive	Regular	Married. Lives with husband, 1 child, 6m.
Alma	White British	Somewhat active	Inactive	Married. Lives with husband and 2 children, 4 and 16m.
Ruby	White British	Inactive	Regular	Married. Lives with husband and 3 children, 8, 5, 18m.
Tamsin	Black British	Inactive	Regular	Lives with male partner, 1 child, 3m
Sula	Somali	Regular	Regular	Married. Lives with husband and 4 children, 3, 6, 9, 11.
Suma	Somali	Regular	Regular	Married. Lives with husband, 1 child, 2 years.
Winnie	Sudanese	Regular	Regular	Married. Lives with husband and 2 children, 5 (with disabilities), 11m
Mika	Black Polish	Inactive	Inactive	Single, lives with husband and 2 children, 3.5 years, 17
Mona	Pakistani	Low	Regular	Married. Lives with husband and 2 children, 3 and 7 (disabilities)
Kiki	Caribbean British	Inactive	Low	Single. Lives with 2 children, 8 and 11 months
Sami	Somali	Low	Low	Single. Lives with 3 children, 3, 6, 7.
Rav	Sudanese	Inactive	Low	Married. Lives with husband and 1 child, 13m

Recruitment was facilitated via the first author’s connections with Children’s Centres, which are situated in the most deprived parts of a UK city, and with which all the participants had a strong, trusted relationship. Potential participants were identified through their use of food clubs serving food to insecure families. These are situated in Children’s Centres, and Children’s Centres’ staff approached users to ask if they were interested in taking part in the research. From the pool of volunteers, postcodes were subsequently checked as aligning with the 10% and 20% most deprived areas of the UK. Furthermore, a maximum variation theoretical sampling technique was also deployed, based on differences in ethnicity and family structure (Patton, 2002). All participants were heterosexual and had at least one pre‐school child. In order to explore the problems of recruitability to LTPA and the patterns of LTPA normative for this group, the majority of participants reported that they had been predominantly inactive since becoming mothers, irrespective of the pandemic disruption that further enforced their sedentariness. However, many had been active prior to motherhood in a range of leisure fields, including gym classes, dancing, running and cycling. Around half the participants were in part‐time paid employment prior to lockdown, but none had continued their employment during the pandemic, although in some cases their male partners were continuing to work. Participants received £25 as a token of appreciation.

Following institutional ethical approval, interviews were conducted online, mainly by a research assistant (RA) with expertise in conducting qualitative social research with women living in areas of deprivation. The RA also lived in one of the more deprived parts of the city and is a mother, as is the first author, who conducted the remainder of the interviews. The researchers’ experiences enabled them to build rapport with participants, and the RA’s experience reduced problematic power dynamics. Online interviews may have created limitations for those digitally excluded, but were necessary due to COVID‐19 social distancing rules and also offered convenience for mothers with caring responsibilities unable to leave the home due to school and childcare closures. Interviews followed a piloted topic guide that followed the ‘funnel’ method (McCracken, 1988), starting with broad questions about experiences of mothering and moving towards a more granular insight into experiences and feelings about LTPA in the past and future. Interviewees knew that the topic of the research was physical activity, which may have led to an emphasis on a desire for LTPA in participants’ lives. However, many had been active prior to motherhood. Furthermore, researchers maintained a reflexive awareness during the interview, helping to ensure participants’ reflections were not prompted by the question framing (Green & Thorogood, 2013). Interviews lasted between 40 and 80 min, were recorded, transcribed and analysed using NVIVO12.

Following guidance on reflexive thematic analysis (RTA) (Braun & Clarke, 2019; Terry & Hayfield, 2020), data familiarisation preceded coding. In line with the principles of theoretical knowingness and transparency central to RTA (Braun et al., 2019), it is important to recognise that researchers engaged with data through the lens of practice theory (Braun & Clarke, 2020), understanding practices as the building blocks of everyday life. Furthermore, researchers had an open interest in inequality based on their prior work with lower‐income mothers (Spotswood et al., 2021) and with women in relation to physical activity (Gurrieri et al., 2013). However, there was no pre‐defined analytical framework. Initial codes recognised patterns of physical activity participation in participants’ pasts, their experiences of lockdown and their future desires and recruitability to envisioned physical activity practices. For example, semantic codes included details of past physical activity and failed attempts, as well as ‘nervousness’ and ‘anxiety’ about future plans to exercise, as well as ‘exhaustion’ and ‘childcare’ acting as obstacles to recruitment. Initial codes were reviewed, and collaborative decisions about useful theorisation of data were made through discussion and through re‐reading extant theoretical research (Byrne, 2022). This process involved authors’ ‘reflective and thoughtful engagement’ (Braun & Clarke, 2019, p. 594) with the data and analytical process, through which initial themes were generated. These were based on researchers’ understandings of meaningfulness in relation to the organising concepts of inequality and capability, and also based on participants’ reflections, repetitions and conviction. As such, both inductive and ‘broad’ deductive coding were adopted (Braun & Clarke, 2020). Furthermore, given the practice theoretical focus of the research, latent coding was deployed alongside semantic coding to develop insights relevant to the research question. Authors moved back and forth through coding stages to eventually generate the capabilities framework presented below (Byrne, 2022).

## TEMPORAL, ENERGY AND SUPPORT CAPABILITIES

**FIGURE 1:**
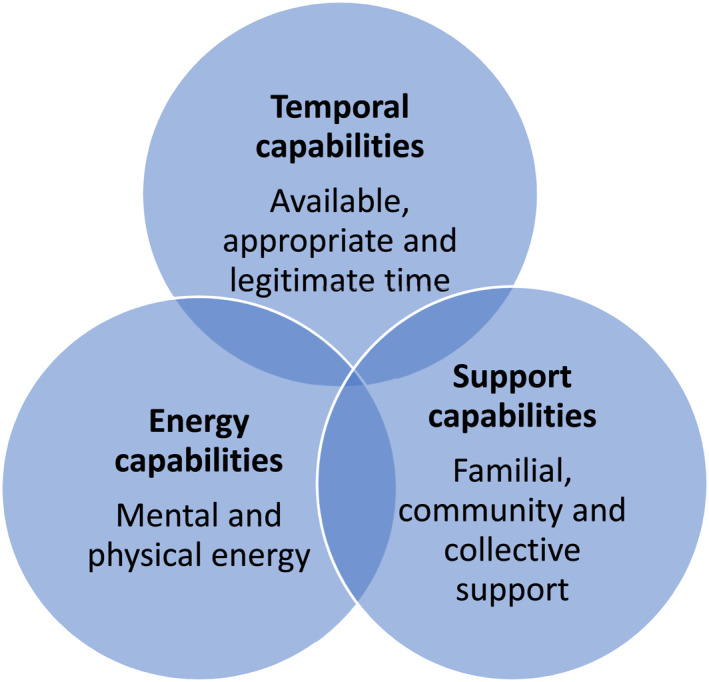
Capabilities necessary for recruitment to LTPA practices for low SES mothers

Many of our participants had engaged in regular LTPA before becoming mothers, and although most had not participated much, or at all, since having children, they recognised the mental, emotional and physical benefits, particularly in the context of the claustrophobic, distressing experience of the COVID‐19 lockdowns. Furthermore, these participants recognised that there were opportunities for LTPA in their areas under normal circumstances. Indeed, some participants had been able to continue their participation using local opportunities. Nonetheless, in the interviews, those less active participants would fantasise about a future in which they spent time going to the gym or joined a running club. For example, Ruby explains that before she was pregnant she went to the local gym and ‘loved it’ adding that ‘if there was something like that on an evening again, then I would try and make that, like, something I did’. However, despite this potential availability and a desire for an aspirational version of motherhood that includes active leisure, the particular configuration of practices that organise the daily lives of our participants is dominated by home and child caring practices that bring strongly gendered normative conventions (Swidler, 2001). In this context, our analysis identified that temporal, support and energy capabilities are necessary for this group of mothers to be recruitable to LTPA practices (see Figure 1). The requirement for these capabilities is compounded by their lower socioeconomic status and related limited financial and social resources that might otherwise enable recruitability. For many of our participants, recruitment to LTPA practices remained problematic, constrained or impossible.

### Temporal capabilities

Temporal capabilities are necessary for LTPA participation because, despite practice availability and practitioner desire, successful enactment of physical activity practices means they must be compatible in the sequencing of everyday life (Southerton, 2013). In this sense, time is a resource required for recruitment. LTPA practices need particular time availability; when practitioners are awake and other practices are absent or can be flexed and de‐prioritised to allow LTPA to take hold. The need for temporal capabilities is strongly connected with the gender‐normative conventions of parenting, irrespective of socioeconomic status. However, financial and social resources available in greater volumes to middle class mothers might help carve out time for LTPA in the form of paid‐for, outsourced childcare opportunities for active leisure with children or during the working day of an autonomous, flexible job. Nonetheless, for most mothers, time is scarce, is fragmented, and also rigidly patterned around other fixed timetables. Furthermore, expectations about how mothers should spend their time did not tend to include LTPA.

Many participants noted repeatedly that they had little time for any leisure now they are mothers. As Kiki explains, ‘I wish I had time for myself… There’s a lot of things I wish I was doing’. Characteristically, Diana explains how her time is spent:

By the time I’ve done everything with the boys in the day, or in the house, or anything like that, I just don’t have the time to do [physical activity].

Jemima also noted that she ‘would like to have time to go to the gym. I’d like to have my own time; a routine, like every Wednesday I go to the gym or going to the swimming pool, enjoying time for myself’. However, she explains that having this time is an ambition rather than a prospect. For Mona, despite having participated in regular gym sessions before having children, even ‘running for hours’ on the treadmill during Ramadan, she has consistently failed to get back to her active routine since becoming a mother:

I’m thinking to join the gym for seven years… Since Mo was born, “next month I will have gym membership, next month I will do that…” [shakes head].

Mona has found it impossible to fit LTPA back into her routine as a mother. Similarly, Kate found that having a second child meant, in her words, ‘the end’ of having time to do anything herself: ‘I fell pregnant with my second son, and I just haven’t done anything since then… you never get that chance’. She admits that ‘I always said, ‘I’m going to do it’, and I just never did’ with reference to getting back to the gym.

Although time is necessary for all potential recruits of LTPA, a lack of time for themselves is compounded for mothers by the highly rigid nature of mothering practices, which demand presence at particular places at particular times (Southerton, 2013). Mothering practices are difficult to flex around active leisure opportunities, described elsewhere as temporally rigid and hostile to LTPA (Spotswood et al., 2021). This temporal rigidity comes from the intersection of many mothering practices at the same time, forming ‘hotspots’ at stressful points of the day. For example, preparing children’s meals, bedtime routines and the ‘school run’ tend to converge with a range of other practices with fixed institutional timetables, such as working hours (of both parents), the opening hours of childcare settings and schools and children’s sleep patterns. Vanessa describes her routine as ‘structured’, and Jemima and Sula both describe having a ‘strict routine’ each day. Jenny notes that ‘your time’s not your own… Things that you wanted to do, you could just do before. Your time was your own.’ For Dora, the rigidity of mothering and the relentless busy‐ness it entails can feel like ‘a nightmare’ and like she’s on ‘a time loop’ preventing her from taking part in any leisure activities for herself:

I What do you do most in your week?

R Um, taking care of everybody else. A lot of pick‐ups. I felt like that is my life. You’re on a time loop basically and I didn’t fit in anything that I wanted to do really.

Even participants who attempted to fit regular LTPA into their busy routines found that the rigidity of mothering practices was hostile to LTPA:

yes, so I’m *aiming* to run three times a week about 5K. Each time I’ve got sort of like a rough route that I do… [but] you know, it’s been half term and obviously… so I haven’t. I haven’t done it for the last two weeks, just ’cause I haven’t had the time while it fitted in with all of them. So I was trying to do it while Margot was asleep. But then [my husband] would be in a meeting, you know?

(Alma)

Alma’s caring practices, her child’s sleep routines and her husband’s working timetable intersect to colonise much of her time in rigid ways, meaning LTPA gets readily pushed out despite her desire to run.

Furthermore, participants described that where leisure time was available, it was fragmented, meaning they still felt they had ‘no time’ for physical activity despite being able to ‘shoehorn’ some other leisure activities into ‘little spaces’ within their daily routine. Here, Amma explains that she can fit in some reading, craft or knitting that take place in the home and in short bursts, but less so physical activity that requires being away from the home for longer periods:

I guess I always feel like I haven’t got time to do “me stuff”. Yeah, so like I don’t really—I don’t—I do a lot but I always feel like I could do more for me. I was going to the gym maybe three to four times a week [before children]. But then I also like to, in my spare time, when I have spare time, make clothes and stuff like that. I just feel like things like that always get pushed to the wayside. Like reading, I might be able to get some in before I fall asleep or that sort of thing. I do manage to always snatch a bit of time to knit…

(Amma)

This fragmentation of mothers’ leisure time, noted elsewhere (Lewis & Ridge, 2005), does not work so well for leisure time physical activity, particularly out‐of‐home classes and meet‐ups. As Amma explained, ‘I find group activities where there’s a start time and a finish time really hard to get to. It’s just difficult. Things like that, it’s just too hard’.

The constraints of available and appropriate time for LTPA are further compounded by collective temporal dispositions towards legitimate ‘use’ of time for mothers (Southerton, 2013); shaped by a traditional heteronormative ideal of parenting that sees women as the dominant provider of care. Many of our participants admitted to the low priority that LTPA had in their lives. Characteristically, and in line with research illuminating the emphasis on ‘intensive mothering’ in Western cultures (Hays, 1996), Kate explains that she does not *want* to leave her children, despite having a supportive ex‐partner who would allow her time for leisure:

I don’t like leaving them. I like to be around at bedtime. I feel that I’m the main person who should be responsible for them so, any time that I do get to myself, I don’t feel like it should be spent doing exercise, when really… probably should! I end up doing washing, or tidying up the house, or thinking about Christmas, and things like that.

(Kate)

Similarly, like many other participants, Jemima emphasises that having children is ‘rewarding’ and ‘a good swap’ for the inevitable exhaustion, but she explains that mothering has pushed out self‐care and self‐development practices as well as LTPA: ‘I could do more things to, like, develop myself, like find a different and better job’. In this line, Tara admits that ‘as mothers, it’s easy to forget about ourselves’ when it comes to prioritising time for themselves, and DS admits that she has ‘never given myself time’ and this ‘spirals my anxiety’. Jenny notes that ‘exercise is probably the first thing that gets pushed to one side when women have children’, acknowledging the low priority that it holds. Kate admits that LTPA is ‘not part of my routine already. There’ll be a time where your kids are grown up and there’s time for it’. Time for their own self‐development and self‐care, including LTPA, was not a priority. As such, available, appropriate and legitimate time constitute the temporal capabilities necessary for LTPA participation, yet were recognised as predominantly absent for our participants, which constrained their recruitability to LTPA practices.

### Support capabilities

For our participants, support capabilities are necessary for LTPA participation because successful recruitment and enactment are dependent on familial, community and collective support. For our lower SES mothers, these were often absent, making their recruitment to LTPA difficult or impossible and compounding a collective understanding that LTPA was not normative for people like them despite their fantasies. Although some participants had family living locally, our sample included immigrant mothers with no local family, single mothers with no partner or family for support, some who were unwilling to engage with the local community and make friends because they felt unsafe due to personal experiences of domestic violence, some whose children’s disabilities made childcare difficult to arrange and afford and some who struggled to integrate because they felt they did not fit in due to cultural or ethnic differences. For example, Kiki (who is black and Caribbean British) explains ‘There’s mostly white people [around here]. I don’t feel safe in this area because I haven’t grew up there and I don’t know no‐one around that area. It’s weird’. Similarly, Mona described being the ‘only Muslim woman’ on the school run and struggling to make friends:

I’m the only Muslim woman and my son is the only Muslim boy in his class so all are English and if I talk to them they are really nice but there is some, I would say, discrimination or something which they don’t show but you can see it. My son is in year two but no mums are my friend so they have their distance. I wouldn’t say it’s a racist area… but they don’t get involved. They’re in their own culture.

(Mona)

Other research studies have noted that women’s access to leisure is dependent on others (McGannon et al., 2018), and our participants were particularly reliant on the support of others given that paid childcare was often beyond their financial means, particularly before children became eligible for part‐time hours of free childcare (at 3 years old, in the UK system). Although some of our active participants explained that they were grateful for their husbands for their support, many of our participants did not have an adequate support network to enable them to participate in regular LTPA. As Amma noted, ‘It’s too much organising the childcare and stuff to do [group physical activity]. It’s just too difficult, things like that. It’s just too hard’.

For our participants, finding support for childcare could be impossible, meaning time constraints limiting their capacity for LTPA could not be easily overcome. Most participants cited ‘lack of childcare’ as the reason for low LTPA participation levels amongst people they knew. As Suma explains, lack of support limits recruitment, despite desire and availability: ‘I don’t know what can be changed because sometimes even if you talk to the ladies and they *want* to do something, what they ask you back is who’s look after the kids?’

Many participants noted that the problem of finding support for LTPA was made harder by the social expectations about the division of labour in couples, families and communities. Sami, a Somali immigrant who considers herself part of the local Somali community, emphasised that although she herself has a supportive husband who will work flexibly to allow her to play basketball one evening a week, for many of her friends, childcare is a major barrier. She emphasises repeatedly in the interview that it is not normative for Somali men to support their wives in taking part in active leisure:

Some ladies they are even, they have partners but the partners if they don’t want to look after the kids, so the mothers they don’t have no choice to do any activity because it’s her responsibility, the men doesn’t want to do, so she stays at home and she forgets the things that she wants to do for herself.

(Sami)

Sami explains that in what she calls ‘the Somali community’, all home‐ and child‐caring practices, including organising additional tuition, are understood as the remit of women:

So all the time you have to do the best like… like bring children to school, bring back from the school, the school run. You have to do the cooking, the cleaning, the shopping, a lot of things. So even the tuition that’s extra thing for Somali ladies because if the child is behind and you think your child is not doing enough at school, they take children to tuition after school time. So even they don’t have no time at weekends because they have to bring children to tuition. So that’s not even one hour for the mother.

(Sami)

Similar reflections about there being ‘not 1 hour for the mother’ were made by other participants of all ethnic backgrounds. Characteristically, Diana explains that she is the ‘main person’ who organises the family schedule, despite being married: ‘I work my day around kind of everybody else. I don’t get an hour to myself’. Similarly, Rebecca summarises how her life is focussed entirely on the children, with little support:

I think I used to do a lot more [physical activity] before I had the children. Because now I’ve got them like 24/7 so I don’t really go out and do exercise as such. I take them out quite a lot. But yeah, I think I was more active before.

Rebecca is married but is nonetheless with the children 24/7. Similarly, Jenny explains the normality of mothers she knows not being able to participate in LTPA due to the lack of support:

I don’t think I’m really that surprised to be honest. I mean, I think it’s harder for women get back into doing things. I think they end up doing, obviously… ’cause they most of the time they got their maternity leave, then being responsible for the childcare. Most of the time there is harder to arrange alternative childcare to go and do different exercises and stuff.

Ruby, also married, explains that she never went back to the gym after having children, despite ‘loving it’, because ‘I didn’t have anyone to have [my daughter]’. She did not mention the cost of the gym, and although gyms can be expensive in the UK, various cheaper community options are available. This suggests that her LTPA was not prioritised or supported. Winnie, concludes that ‘Men are working, come late and that is most happening. Women are left behind long, long ago’.

The expectations about mothers being ‘present to care’ for their children (Spotswood et al., 2021) and the lack of support for their LTPA participation meant our participants tended not to know others ‘like them’ who participate routinely, and this has further repercussions for lower SES women in terms of the support they need but lack. As Diana admits, although there is a local leisure centre, ‘it’s hard walking in there if you’re not going with anybody else’, emphasising that the potential participation would be solitary and difficult for her given the lack of support for her participation. Similarly, Clara notes that one reason people she knows give up doing LTPA is that ‘They don’t know anybody, and things like that. It’s alright if you’ve got a friend or someone in the same boat as you to go together…’ Connections between potential and existing participants form ‘crucibles’ in which new arrangements might be formed (Shove et al., 2012, p. 67) and which make recruitment to new practices possible (Maller & Strengers, 2013). Lower SES mothers ‘don’t know where to go and what to do’ (Sami) because they do not have the support of others from whom they can ‘learn the ropes’ of new LTPA practices (Shove et al., 2012). For some, the collective understanding of the lack of support for LTPA meant they had internalised a wariness about participating in LTPA, stemming from the certainty that other participants would be unfamiliar:

how do I know there’s not someone [at a physical activity session] that’s not very nice or someone that’s a weirdo? I’d never go if I didn’t know who’d be there.

(Vanessa)

Similarly, Diana explains she would only take part in group physical activity if she has the chance to meet the instructor first and allay her anxieties and ‘what I need to bring and what I should be wearing… they’re the things that worry me’. The lack of familial, community and collective support for LTPA participation means LTPA is pragmatically difficult but also understood to be anomalous, so envisioning participation triggers anxiety amongst participants. The lack of these support capabilities therefore conditions and constrains the possibilities for lower SES mothers’ recruitability to LTPA practices.

### Energy capabilities

Finally, energy capabilities are necessary for LTPA participation because successful enactment of physical activity requires practitioners to have mental and physical energy resources. We define energy as a positive mood state that ‘refers to feelings of having the capacity to complete mental or physical activities’ (O’Connor, 2006, p. s7). If energy is lacking, it means practitioners are ‘more likely to avoid physical or mental work if it is possible to do so’ (*ibid,* p. s9). Sports science researchers have long been interested in ‘low energy’, because it is experienced by a wide range of people (for example, it is the most common complaint expressed during pregnancy) and has been found to be both reduced through physical activity participation and also result from inactivity (O’Connor & Puetz, 2005). ‘In short, feelings of fatigue and energy are strongly associated with health and quality of life’ (*ibid*, p. 299). In this line, our participants found that their everyday lives were coloured by having low mental and physical energy. Particularly, participants understood their low energy to come from the overwhelming demands that mothering practices made of their time, but also their physical energy and mental energy resources. Amma explains, for example, that mothering is relentless:

I’m not the person I was before in many ways. [Mothering] is all consuming. It doesn’t stop. It’s constant. There’s always things to do.

Referring to physical energy, some described the feeling that they had become sapped of strength as a result of pregnancy and childbirth. Fran explains that she is ‘not actually able to do what I could do before anyway. So it’s a bit sad’. She further explains:

I have some complications left over from my pregnancy, I’ve got diastasis recti, which is the tummy gap, the muscles haven’t joined back in my stomach after pregnancy. So actually, I have very little core strength now anyway.

Pregnancy and childbirth can be a drain on mothers’ physical energy, but so can everyday mothering, given the heavily physical tasks it involves. For example, Diana responds to a question about how she spends her time with, ‘Carrying a 10 month old and feeling tired [laughs]’. Similarly, Kate describes what she does most in the week as ‘I chase after the boys. That’s not physical activity for me—that’s just “Mum‐duty”’. Similarly, Vanessa answers questions about whether she is active with ‘It’s called running round after a two and a half year old…’ Mothering practices can be physically draining, taking a toll on the energy levels necessary for recruitment to active leisure.

Our participants also described the particular mental energy demands of mothering and described the lack of mental energy for other activities. Tia describes feeling strained every day since becoming a mother:

Mentally, physically [I feel different]. I mean he still doesn’t sleep through the night so I’m strained every day.

(Tia)

Tia feels ‘strained’ compounded by the poor sleep she routinely gets. The issue of poor quality sleep was noted by most of the mothers, highlighting the extent to which the extensive and rigid temporal demands of mothering practices impact both physical and mental energy levels. Similarly, Tamsin describes the ‘constant grogginess’ that characterises her experience of mothering:

I get far less sleep, well I say I get far less sleep, I get a continual lack of sleep whereas [before] I was quite used to doing night shifts, but it’s a different type of sleep deprivation. So it’s a grogginess that just has lasted for months, rather than a short sharp burst and then you recover. Life revolves around my little one now, so it’s not about when I can do things when I want to, it’s about what works for her.

In addition to, and compounded by, poor quality sleep, participants described the mental focus required to care for small children. Vanessa describes the unremitting focus that mothering requires: ‘With having a little one already you have to make sure you’re watching them constantly’. Similarly, Josie feels she cannot relax at any point when she is with her small children:

I feel I have to watch them all the time. Both of them would happily run in the road and not think about it. So, the entire time − you feel more frantic, I suppose, because you’re concentrating on a million things. That’s what it’s like with them.

Our participants describe having ‘nothing left for me’ (Amma), referring to their low energy. For example, Alma describes feeling ‘lethargic… you just get that whole ‘I can’t be bothered’ attitude, you know, just being bothered’. Tamsin describes feeling exhausted by 8 PM. She explains ‘To be honest I’m shut down’. Similarly, Mika does not feel she has the energy she used to, which she feels when she tries to go shopping:

I can feel it now. I don’t have that much energy like I had before. Even when I go shopping I’m so tired… especially for my back. I have the back pain because I’m not doing much anymore so definitely… I just have a bag that you can put on your arm and you come back from there carrying not much and you just feel so tired.

Mika feels that mothering has sapped her energy.

Unsurprisingly, our participants described their low energy levels as a key reason they failed to pursue opportunities for LTPA that have arisen in the past and why they struggle to imagine taking part in the future. For example, Kiki likes the idea of LTPA but feels like she is not in the ‘right mood’ for it:

I struggle with working out—I don’t feel in the mood for it—I can’t explain. I love to go running but, because I’m so busy—non‐stop kids and everything—I don’t really… It doesn’t even cross my mind anymore—it’s terrible. But I would love to exercise again.

(Kiki, 3)

Kiki has lost the drive to run, despite her love of running, because of the low energy she feels from her everyday life of ‘non‐stop kids’. Kate also explains her lack of energy as constraining her ability to join in with LTPA, despite a tangible opportunity:

as soon as I get five minutes, I’m doing housework or shopping—you don’t actually do anything for yourself. My Mum works for a gym—she’s trained as a personal trainer—and I actually signed up to the gym, and I said to her, I can come up with her, I can do it on the nights that she’s working, but I just… By the time I’ve done everything with the boys in the day, or in the house, or anything like that, I just don’t have the time to do it—I don’t feel like doing any of it, to be honest.

Kate is a single mother and feels that every moment is accounted for by home and childcaring practices, with no time for herself, and no energy left for herself either. She adds that she does not have time to exercise, is tired and feels she ‘can’t be bothered’:

I just don’t have the… I *do* have the time, I don’t make time to do it—I know that. I’m tired—that is the only thing that I can put it down to—I’m tired, and I can’t be bothered most days.

Kate’s constant ‘Mum‐duty’ is draining and leaves her with insufficient energy to contemplate integrating LTPA into her everyday life, despite the time and opportunity. This was a similar experience for Jemima, who describes her low energy as a result of juggling three jobs and three children on her own. She describes her children asking her to participate in their games, but she simply cannot muster the energy:

once again they’re already riding their bikes in the park and they’re asking me if I want to try, but I say “no it’s fine”. I regret it afterwards and I think I should have done it… but I’m feeling like a really old person who is sitting at home cooking and washing [laughs].

Jemima adds that she would ‘love to have the motivation to go jogging or running’ and refers to herself as ‘lazy’. A number of other participants also blamed themselves for their low energy, which they interpreted as deficient motivation. Tia, for example, felt that her low motivation was her own fault, commenting that ‘I feel like I could do a bit more but it’s just finding the time and the motivation really’.

Energy capabilities in the form of physical and mental energy are necessary to become recruitable to LTPA, and to condition the extent to which LTPA becomes envisioned as possible and appealing. Low SES mothers lack physical energy due to the nature of mothering practices that dominate so much of their lives, due to the mental demands of caring for young children and their lack of opportunity for a break. The lack of energy capabilities constrains our participants’ retractability to LTPA practices.

## DISCUSSION

**FIGURE 2:**
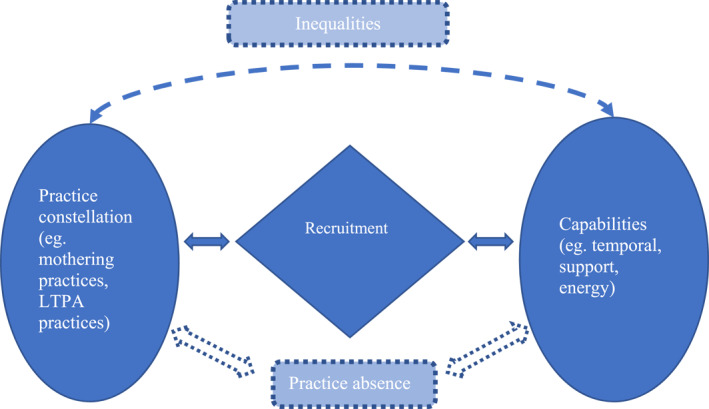
Practice capabilities and health inequalities

In this article, we have explored how a capabilities perspective can advance practice theoretic conceptualisations of persistent health inequalities. Our participants exhibit a ‘desire’ for the routine incorporation of LTPA into their lives, although we acknowledge that participating in physical activity represents a powerful archetype driven by government public health and private marketing and communications that responsibilise people to perform health risk management behaviour. Our research sets out to understand the capabilities that form an important part of the unequal picture of recruitability to leisure time physical activity (LTPA) practices for low SES mothers—a group that has traditionally been excluded. In line with other studies that describe the inequalities between men and women’s engagement with leisure (Bittman & Wajcman, 2000), our study illuminates that our lower SES participants, who are mothers, face complex constraints on their recruitability to LTPA practices despite, in many cases, the availability of practice elements. These constraints can be particularly exacerbated by lower socioeconomic status that places the resources necessary for alternative routes to recruitment out of reach, but we follow Sen and other feminist readers of the capabilities perspective in refusing to collapse inequality into matters of resource availability (Robeyns, 2003; Sen, 1992). Through our study, we illuminate how temporal, support and energy capabilities are necessary for low SES mothers to integrate the elements necessary for the performance of LTPA, contributing to practice theory research that assumes the availability of practice elements is sufficient. The dispossession of these intertwined capabilities signals inequalities emerging from the constellation of practices that configure this group’s lived experience as mothers, in turn giving rise to practice absence and further consolidating patterns of inequality across gender and socioeconomic lines. We depict this in our framework of practice capabilities and health inequalities (Figure 2) and next discuss three key contributions to conceptualisations of practice recruitment, inequalities and absence in relation to health.

First, we highlight the utility of a capabilities perspective for problematising extant conceptualizations of recruitment. Recruitment to practices is often presented as simplistic and unproblematic (Denegri‐Knott et al., 2018; Walker, 2015) yet researchers have highlighted that practitioners’ experiences, abilities and capacities can affect how they access and integrate the necessary materials, meanings and competences for practice enactment (Maller, 2019), negotiating and even rejecting the practice template (Molander & Hartmann, 2018). Building on these ideas, we draw on the capabilities framework (Sen, 2005, 2009) to illuminate how capabilities impose conditions on practitioners’ integration of practice elements. We contend that capabilities differentiate how practitioners come to participate in practices—and may, in fact, obstruct recruitment. In highlighting the constraints on recruitment and the ultimate failure of LTPA practices to recruit some population segments, we contribute empirically and theoretically to the tendency for practice‐theoretic repertoires to elide problematising recruitment (Walker, 2015). For our participants, recruitability to LTPA was often not a matter of available elements, but a lack of the capabilities necessary to integrate these in successful LTPA performance. In bringing this perspective to practice‐oriented sociology of health and illness research, we show that capabilities are also necessary for the integration of elements in health‐promoting practice performances, and therefore for the patterned trajectories that practices follow.

In advancing this perspective, we respond to the call by Halkier and Holm (2021) for a ‘differentiated’ practice approach to health research because the capabilities necessary for recruitment to health‐promoting practices connect with unequal practice constellations that characterise the collective experiences of different groups. As such, our second contribution is to advance a conceptualisation of inequalities in practice performance through a capabilities lens. Our capabilities perspective foregrounds practitioner lived experiences of exclusion, yet theorises these as conditions of past and present practices. Our study illuminates how the capabilities necessary for low SES mothers’ recruitability to LTPA are both interconnected and contingent on mothering practices, which are shaped by gendered and heteronormative cultural conventions that limit participants’ capacity to integrate available elements and become recruitable to LTPA (Robeyns, 2003). Temporal constraints limit the pragmatic possibilities of sequencing LTPA alongside mothering practices that are often held fixed by institutional routines and timetables as well as culturally normative expectations about the mothers’ role in the family (Spotswood et al., 2021). Although ‘desired’ at a fantasy level linked to cultural archetypes of health risk management and appearance management in light of the male gaze (Gurrieri et al., 2013), routinely enacted LTPA manifests as a low priority for participant partners, families and communities. Although some of our participants had poor support networks because they were actively reclusive or felt disconnected from their communities, others described a lack of support as emerging from the low priority placed on women’s active leisure time in their communities. In these ways, temporal and support capabilities are entangled, reconstituting each other and closely tied to the strength and narrow cultural ideals of gender‐normativity. Furthermore, the limited support our participants had for leisure compounded their low energy. Low energy also emerged from the temporal demands of mothering, the poor sleep often brought by caring for young children and also the physical demands of pregnancy and childbirth. These three capabilities are entangled, creating an entrenched texture of exclusion for low SES mothers. Moreover, in our study, mothering practices loomed large in constraining the temporal, support and energy capabilities needed to recruit practitioners to LTPA. This demonstrates how existing and historical patterns of social practices that characterise collective experiences condition the availability of capabilities but also create the conditions for their necessity. This link attends to an important gap, namely theorising and accounting for inequalities in practice recruitment and performance that help explain inequalities in health‐promoting practices across populations. In our article, we have particularly illuminated how gender inequality, compounded by socioeconomic status, becomes a question of ‘disparate freedoms’ (Sen, 1992, p. 125) to access LTPA.

Finally, our framework signals an important but neglected area of practice theorisation—both in the sociology of health and illness and also in practice theory more generally—practice absence. Patterns of practice absence are priority considerations for national health agendas, but are not well mapped or understood in extant practice‐oriented health research. Yet, the range of practices *not* enacted is as significant as those that are. Our framework points to how capabilities constrain and enable recruitment, linking capabilities with practice absences. Furthermore, practice constellations comprise, and are shaped by, practice absence, in that where constellations offer opportunities for capability accrual, practice integration and performance become available. In our research, practice constellations are dominated by the presence of mothering practices and the absence of LTPA practices but other practices ‘crossing through’ low SES mothers (Reckwitz, 2002) and absent from their lives also contextualise the availability of capabilities. Hence, our research points to the importance of capabilities in understanding both practice enactment and practice absence, whereby capabilities are made available or not by the constellation of practices that configure lived experiences and these condition future practice potentialities and therefore practice trajectories.

To conclude, in this article, we advance existing practice theory health and illness research that has largely glossed over questions of social difference in considering unequal performances of health‐promoting practices by different groups (Maller, 2016, 2019) or identified the distribution or availability of practice elements as explaining inequalities (Ally et al., 2016; Blue et al., 2016). This represents an area of insufficient theoretical development in practice‐oriented health research, which is especially problematic given the pervasive inequalities in population health and the emphasis on health inequalities in health research and policy. For example, there is established evidence that LTPA reduces when women become mothers, especially those from lower socioeconomic backgrounds (Bellows‐Riecken & Rhodes, 2008; McGannon et al., 2018). Leaning on the inherent conceptual benefits of practice theory, that refuses to privilege wider determinants or responsibilise individuals in understanding and challenging persistent societal problems in health (Supski et al., 2017), our framework expands understanding of how practice‐theorised health intervention and policy might be focused. Practice theory holds that individual health is ‘the outcome of recruitment to certain practices and not others’ (Maller, 2015, p. 76) so the ‘lives of social practices’ should be the targets of analysis and intervention (Blue et al., 2016, p. 38). However, extant research has insufficiently accounted for the conditions necessary for recruitment to these certain practices. Future policy should focus on both practice configurations and capabilities as conditions for recruitment, thereby laying the foundations for a differentiated practice‐oriented public health that can account for persistent ‘inequities of access’ (Shove et al., 2012, p. 65) to health‐promoting practices and also theorise experiences of inequality, absence and exclusion. We call for future research to unpack the possibilities for policymakers in understanding the nexus of capabilities, practice constellations and practice absence in health and illness research, yet without excluding the experiences, perspective and emotions of excluded practitioners.

## AUTHOR CONTRIBUTIONS

**Fiona Spotswood**: Conceptualization (Lead); Data curation (Lead); Formal analysis (Lead); Funding acquisition (Lead); Investigation (Lead); Methodology (Lead); Project administration (Lead); Resources (Lead); Writing – original draft (Lead); Writing – review & editing (Lead). **Lauren Gurrieri**: Conceptualization (Supporting); Formal analysis (Supporting); Writing – original draft (Supporting); Writing – review & editing (Equal).

## ETHICS STATEMENT

This research was conducted with approval from the University of Bristol School of Management ethics committee (management‐ethicscommittee@bristol.ac.uk).

## Data Availability

Author elects to not share data—Research data are not shared.

## ENDNOTE

^1^

https://www.facebook.com/NHSwebsite/videos/better‐health/369814797315611/
